# A study of transformer-based end-to-end speech recognition system for Kazakh language

**DOI:** 10.1038/s41598-022-12260-y

**Published:** 2022-05-18

**Authors:** Mamyrbayev Orken, Oralbekova Dina, Alimhan Keylan, Turdalykyzy Tolganay, Othman Mohamed

**Affiliations:** 1 Institute of Information and Computational Technologies CS MES RK, Almaty, Kazakhstan; 2 Satbayev University, Almaty, Kazakhstan; 3 L.N. Gumilyov Eurasian National University, Nur-Sultan, Kazakhstan; 4 Universiti Putra Malaysia, Kuala Lumpur, Malaysia

**Keywords:** Computer science, Information technology, Scientific data

## Abstract

Today, the Transformer model, which allows parallelization and also has its own internal attention, has been widely used in the field of speech recognition. The great advantage of this architecture is the fast learning speed, and the lack of sequential operation, as with recurrent neural networks. In this work, Transformer models and an end-to-end model based on connectionist temporal classification were considered to build a system for automatic recognition of Kazakh speech. It is known that Kazakh is part of a number of agglutinative languages and has limited data for implementing speech recognition systems. Some studies have shown that the Transformer model improves system performance for low-resource languages. Based on our experiments, it was revealed that the joint use of Transformer and connectionist temporal classification models contributed to improving the performance of the Kazakh speech recognition system and with an integrated language model it showed the best character error rate 3.7% on a clean dataset.

## Introduction

Innovative information and digital technologies are increasingly making their way into the life of a modern person: this applies to deep learning systems like voice recognition, images, speech recognition and synthesis. Namely, speech technologies are widely used in communications, robotics and other areas of professional activity. Speech recognition is a way to interact with technology. Speech recognition technology provides recognition of individual words or text, with its further conversion into a sequence of words or commands. There are traditional speech recognition systems that are based on acoustic, language models and lexicon. The acoustic model (AM) was built based on hidden Markov models (HMM) with the Gaussian Mixture Model (GMM), and the language model (LM) was based on n-gram models. The components of these systems were trained separately, which made it difficult to manage and configure them, which led to a decrease in the efficiency of using these systems. With the advent of deep learning, the performance of speech to text systems has improved. Artificial neural networks began to be used for acoustic modeling instead of GMM, which led to improved results that were obtained in many research works^1–3^. Thus, the HMM-DNN architecture has become one of the most common models for continuous speech recognition.

Currently, the end-to-end (E2E) model has become widespread. The E2E structure presents the system as a single neural network, unlike the traditional one, which has several independent elements^4,5^. The E2E system provides direct reflection of acoustic signals in the sequence of labels without intermediate states, without the need to perform subsequent processing at the output, which makes it easy to implement. To increase the performance of E2E systems, it is necessary to solve the main tasks related to the definition of the model architecture, the collection of a sufficiently large speech corpus with the appropriate transcription, and the availability of high-performance equipment. Solving these issues ensures the successful implementation of not only speech recognition systems, but also other deep learning systems. In addition, E2E systems can significantly improve the quality of recognition from learning large amounts of training data.

Models based on the Connectionist temporal classification^6^ (CTC), models based on the attention mechanism^7^ are illustrative examples of end-to-end systems. In a CTC-based model, there is no need to align at the frame level between acoustics and transcription, since a special token is allocated, like an "empty label" which determines the beginning and end of one phoneme^8^. In the attention mechanism based encoder/decoder models, the encoder is an AM—converts input speech into a high-level representation, the attention mechanism is an alignment model, and determines encoded frames that are related to the creation of the current output, the decoder is similar to the AM—operates autoregressive, predicting each output token depending on previous predictions^9^. The above E2E models are based on convolutional and modified recurrent neural networks (RNNs). The models implemented using RNN perform calculations on the character positions of the input and output data, thus generating a sequence of hidden states depending on the previous hidden state of the network. This sequential process does not provide parallelization of learning in training examples, which is a problem with a longer sequence of input data and takes much longer to train the network. In^10^, another Transformer-based model was proposed, which allows parallelization of the learning process, and this model also removes repetitions and uses its internal attention to find the dependencies between the received and resulting data. The big advantage of this architecture is the fast-learning rate and the lack of sequential operation, as with RNN. In previous studies^11,12^ it was revealed that the combined use of Transformer models and an E2E model, like CTC, contributed to the improvement of the quality of the English and Chinese speech recognition system.

It should be noted that the attention mechanism is a common method that greatly improves the quality of the system in machine translation and speech recognition. And the Transformer model uses this attention mechanism to increase the learning rate. This model has its own internal attention, which aligns all positions of the input sequence to find a representation of the set, which does not require alignments. In addition, Transformer does not need to process the end of the text after processing its start.

In order to implement such models, a large amount of speech data are required for training, which is problematic for languages with limited training data, namely for the Kazakh language, which is included in the group of agglutinative languages. To date, systems have been developed based on the CTC model^13,14^ for recognizing Kazakh speech with different sets of training data. The use of other methods and models to improve the accuracy of recognition of the Kazakh speech is a promising direction and can improve the performance of the recognition system with a small size of the training sample.

The main goal of our study is to improve the accuracy of the automatic recognition system for Kazakh continuous speech by increasing training data, as well as the use of models based on Transformer and CTC for recognizing Kazakh speech.

The structure of the work is given in the following order: Sect. 2 presents traditional methods of speech recognition, Sect. 3 provides an analytical review of the scientific direction. Section 4 describes the principles of operation of the Transformer-based model and the model we proposed. Further, in Sects. 5 and 6, our experimental data, corpus of speech, and equipment for the experiment are described, and the results obtained are analyzed. The conclusions are given in the final section..

## Traditional speech recognition methods

Traditional sequence recognition focused on estimating the maximum a posteriori probability. Formally, this approach is a transformation of a sequence of acoustic speech characteristics X into a sequence of words W. Acoustic characteristics are a sequence of feature vectors of length *T: X* = *{x*_*t*_* ∈ R*^*D*^* | t* = *1,*…*, T}*, and the sequence of words is defined as *W* = *{w*_*n*_* ∈ V | n* = *1,*…*, N}*, having length N, where V is a vocabulary. The most probable word sequence *W*^*∗*^ can be estimated by maximizing *P(W|X)* for all possible word sequences *V*^*∗*^ (1)^15^. This process can be represented by the following expression:

1 $$\begin{aligned} & W* \, = \, argmax \, P\left( {W \, | \, X} \right) \\ & \quad W \in V* \\ \end{aligned}$$

Therefore, the main goal of the automatic speech recognition (ASR) is to find a suitable model that will accurately determine the posterior distribution $P\left( {W \, | \, X} \right)$.

The process of automatic speech recognition consists of sequences of the following steps:

- Extraction of features from the input signal.
- Acoustic modeling (determines which phones were pronounced for subsequent recognition).
- Language modeling (checks the correspondence of spoken words to the most likely sequences).
- Decoding a sequence of words spoken by a person.

The most important parts of a speech recognition system are feature extraction methods and recognition methods. Feature extraction is a process that allocates a small amount of data essential for solving a problem. To extract features, Mel-frequency cepstral coefficients (MFCC) and perceptual linear prediction (PLP) algorithms are commonly used^16–18^. The popular one is MFCC.

In the speech recognition task, the original signal is converted into feature vectors, on the basis of which classification will then be performed.

### Acoustic model

The acoustic model (AM) uses deep neural networks and hidden Markov models. Deep neural network, convolutional neural network (CNN), or long short-term memory, which is a variant of the recurrent neural network is used to map the acoustic frame *x*_*t*_ to the phonetic state of the subsequent *f*_*t*_ at each input time *t* (2):

2 $$P\left( {f_{t} |x_{t} } \right) \, = \, AcousticModel\left( {x_{t} } \right)$$

Before this acoustic modeling procedure, the output targets of the neural network models, a sequence of phonetic states at the frame level *f*_*1:T*_, are generated by HMM and GMM in special training methods. GMM models the acoustic element at the frame level *x*_*1:T*_, and HMM estimates the most probable sequence of phonetic states *f*_*1:T*_.

The acoustic model is optimized for the cross-entropy error, which is the phonetic classification error per frame.

### Language model

The language model *p(w)* models the most probable sequences of words regardless of acoustics (3):

3 $$P\left( {w_{u} |w_{ < u} } \right) \, = \, LanguageModel\left( {w_{ < u} } \right)$$

where *w*_<*u*_ is the previous recognized word.

Currently, RNN or LSTM are commonly used extensively for language model architecture, as they can capture long-term dependencies rather than traditional *n-gram* models, which are based on the Markov assumption and limited to a certain n-range of word history.

### Hidden Markov models

For a long time, a system based on hidden Markov models (HMM) was the main model for continuous speech recognition. The HMM mechanism can be used not only in acoustic modeling but also in the language model. But in general, the use of the HMM model gives a greater advantage when modeling the acoustic component.

In this HMM, the phone is the observation and the feature is the latent state. For an HMM that has a state set {1,…, J}, the HMM-based model uses the Bayesian theorem and introduces the HMM state sequence S = {s_t_ ∈ {1,…, J} | t = 1,…, T} пo p (L|X) (4).

4 $$\mathop {argmax}\limits_{{L \in \gamma ^{*} }} ~p\left( {L{\text{|}}X} \right) \approx \mathop {argmax}\limits_{{L \in \gamma ^{*} }} ~\mathop \sum \limits_{S} p\left( {X{\text{|}}S} \right)~p\left( {S{\text{|}}L} \right)~p\left( L \right)$$

p(X|S), p(S|L), and p(L) in Eq. (4) correspond to the acoustic model, the pronunciation model and the language model, respectively.

The acoustic model P (X|S) indicates the probability of observing X from the hidden sequence S. According to the probability chain rule and the observation independence hypothesis in the HMM (observations at any time depend only on the hidden state at that time), P(X|S) can be decomposed into the following form (5):

5 $$p\left( {X{|}S} \right) = \mathop \prod \limits_{t = 1}^{T} p\left( {x_{t} {|}x_{1} , \ldots , x_{t - 1} , S} \right) \approx \mathop \prod \limits_{t = 1}^{T} p\left( {x_{t} {|}s_{t} } \right) \propto \mathop \prod \limits_{t = 1}^{T} \frac{{p(s_{t} |x_{t} )}}{{p\left( {s_{t} } \right)}}$$

In the acoustic model, p (x_t_|s_t_) is the probability of observation, which is usually represented by mixtures of Gaussian distributions. The distribution of the posteriori probability of the hidden state p (s_t_|x_t_) can be calculated by the method of deep neural networks.

Two approaches, HMM-GMM and HMM-DNN, can be used to calculate p (X|S) in Eq. 5. The first approach HMM-GMM was for a long time the main method for building speech-to-text technology. With the development of deep learning technology, DNN is introduced into speech recognition for acoustic modeling. The role of DNN is to calculate the posterior probability of the HMM state, which can be converted into probabilities, replacing the usual GMM observation probability. Consequently, the transition of HMM-GMM to the hybrid model HMM-DNN has yielded excellent recognition results, and is becoming a popular ASR architecture.

Hybrid models have some important limitations. For example, ANN with more than two hidden levels were rarely used due to computational performance limitations, and the context-dependent model described above takes into account numerous effective methods developed for GMM-HMM.

The learning process is complex and difficult for global optimization. Components of traditional models are usually trained on different datasets and methods.

### Hybrid models based on DNN-HMM

To calculate *P(x*_*t*_*|s*_*t*_) directly, GMM was used, because this model gives the possibility to simulate the distribution for each state, allowing to obtain probability values of input sequences. However, in practice, these assumptions cannot always be modeled by GMM. DNNs have shown significant improvements over GMMs due to their ability to study nonlinear functions. DNN cannot directly provide a conditional probability. The frame-by-frame posterior distribution is used to turn the probability model *P(x*_*t*_*|s*_*t*_*)* into a classification problem *P(s*_*t*_*|x*_*t*_*)* using a pseudo-likelihood trick as a joint probability approximation (6)^15^. The application this probability is referred to as a "hybrid  architecture".

6 $$\prod_{t=1}^{T}P({x}_{t}|{s}_{t})= \prod_{t=1}^{T}\frac{P({x}_{t}|{s}_{t})}{p({s}_{t})}$$

A numerator is a DNN classifier trained with a set of input functions as input *x*_*t*_ and target state *s*_*t*_. The denominator P(st) is the prior probability of the state *s*_*t*_. Frame-by-frame training requires frame-by-frame alignment with *x*_*t*_ as input and *s*_*t*_ as target. This negotiation is usually achieved by using a weaker HMM/GMM negotiation system or using human-made dictionaries. The quality and quantity of alignment labels are usually the most significant limitations of the hybrid approach.

### End-to-end speech recognition models

E2E automatic speech recognition is a new technology in the field of ASR based on a neural network, which offers many advantages. E2E ASR is a single integrated approach with a much simpler training approach with models that work at a low audio frame rate. This reduces learning time, decoding time, and allows joint optimization with subsequent processing, such as understanding the natural language.

For the global calculation of *P(W | X)* using E2E speech recognition models, the input can be represented as a sequence of acoustic features *X* = *(x*_*1*_*,…, x*_*t*_*)*, the sequence of target marks as *y* = *(y*_*1*_*,…, y*_*t*_*)*, and the sequences words in the form *W* = *w*_*m*_ = *(w*_*1*_*,…, w*_*m*_*)*.

Thus, the ANN finds probabilities *P(∙|x*_*1*_*),…,P(∙|x*_*t*_*)*, where the input probability parameters are some representations of a sequence of words, i.e. labels.

The basic principle of operation is that modern E2E models are trained on the basis of big data. From the above, we can detect the main problem, it concerns the recognition of languages with limited training data, such as Kazakh, Kyrgyz, Turkish, etc. For such low-resource languages, there are no large corpuses of training data.

## Related work/literature review

The Transformer model was first introduced in^8^, in order to reduce sequential calculations and the number of operations for correlating input and output position signals. Experiments were conducted on machine translation tasks, from English to German and from English to French. As a result, the model was shown to have achieved good performance compared to existing results. Moreover, Transformer works perfectly for other tasks with large and limited training data, and is very fruitful for all kinds of seq2seq tasks.

The use of Transformer for speech-to-text conversion also showed good results and was reflected in the following research papers:

To implement a faster and more accurate ASR system, Transformer and ASR achievements based on RNN were combined by Karita et al.^11^. To build the model, a Connectionist temporal classification (CTC) was E2E with Transformer for co-learning and decoding. This approach speeds up learning and facilitates LM integration. The proposed ASR system implements significant improvements in various ASR tasks. For example, it lowered WER from 11.1% to 4.5% for the Wall Street Journal and from 16.1% to 11.6% for TED-LIUM, introducing CTC and LM integration into the Transformer baseline.

Moritz et al.^19^ proposed a Transformer-based model for streaming speech recognition that requires an entire speech utterance as input. Time-limited self-attention in the encoder and triggered attention for the encoder-decoder with attention mechanism were applied to generate the output after the spoken word. The model architecture achieved the best result in E2E streaming speech recognition − 2.8% and 7.3% WER for "pure" and "other" LibriSpeech test data.

The Weak-Attention Suppression (WAS) method was proposed by Yangyang Shi and other^20^, which dynamically causes sparse attention probabilities. This method suppresses the attention of uncritical and redundant continuous acoustic frames and is more likely to suppress past frames than future ones. It was shown that the proposed method leads to a decrease in WER compared to the basic types of Transformer. In Test LibriSpeech, the proposed WAS method reduced WER by 10% in cleanliness testing and by 5% in another test for streaming Transformers, which led to a new advanced level among streaming models.

Dong Linhao and co-authors^21^ presented a Speech-Transformer system using a 2D attention mechanism that co-processes the time and frequency axes of 2D speech inputs, thereby providing more expressive representations for the Speech-Transformer. The Wall Street Journal (WSJ) corpus was used as training data. The results of the experiment showed that this model allows to reduce the training time and at the same time can provide a competitive WER.

Gangi et al.^22^ suggested Transformer with SLT adaptation—an architecture for spoken language translation, for processing long input sequences with low information density to solve ASR problems. The adaptation was based on downsampling the input data using convolutional neural networks and modeling the two-dimensional nature of the audio spectrogram using 2D components. Experiments show that the SLT-adapted Transformer outperforms the RNN-based baseline in both translation quality and learning time, providing high performance in six language areas.

Takaaki Hori et al.^23^ advanced the Transformer architecture, on the basis of which a context window was developed, which was trained in monologue and dialogue scenarios. Monologue tests on CSJ and TED-LIUM3 and dialog tests on SWITCHBOARD and HKUST were applied. As a result, results were obtained that surpass the basic E2E ASR with one sound and with or without speaker i-vectors.

In the E2E system, the RNN-based encoder-decoder model was replaced by the Transformer architecture in Chang X. et al. research^24^. And in order to use this model in the masking network of the neural beamformer in the multi-channel case, the self-attention component has been modified so that it is limited to a segment, rather than the entire sequence, in order to reduce the amount of computation. In addition to improvements to the model architecture, preprocessing of external dereverberation, weighted prediction error (WPE), was also included, which allows the model to process reverberated signals. Experiments with the extended wsj1-2mix corpus show that Transformer-based models achieve better results in echo-free conditions in single-channel and multi-channel modes, respectively.

## Transformer architecture

The Transformer model was first created for machine translation, replacing recurrent neural networks (RNNs) in natural language processing (NLP) tasks. In this model, recurrence was completely eliminated, instead, for each statement, using the internal attention mechanism (self-attention mechanism), signs were built to identify the significance of other sequences for this utterance. Therefore, the generated features for a given statement are the result of linear transformations of sequence features that are significant.

**Figure 1:**
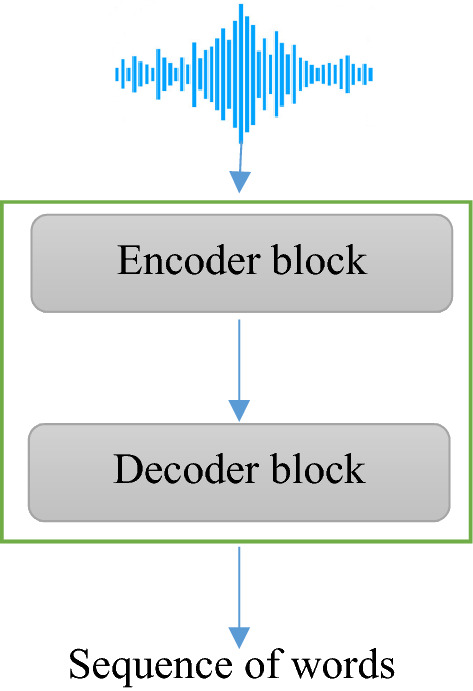
General scheme of the model.

The Transformer model consists of one large block, which in turn consists of blocks of encoders and decoders (Fig. 1). Here, the encoder takes as input the feature vectors from the audio signal *X* = *(x*_*1*_*,…,x*_*T*_*)* and outputs a sequence of intermediate representations. Further, based on the received representations, the decoder reproduces the output sequence *W* = *w*_*m*_ = *(w*_*1*_*,…,w*_*M*_*)*. Each stage of the model uses the previous symbols to output the next, because it is autoregressive. The Transformer architecture uses several layers of self-attention in the encoder and decoder blocks that are interconnected with each other. Consider each block individually.

### Encoder and decoder networks

Conventional E2E encoder/decoder models for speech recognition tasks consist of a single encoder and decoder, an attention mechanism. The encoder converts the vector of acoustic features into an alternative representation, and the decoder predicts a sequence of labels from the alternative information provided by the encoder, then attention highlights the significant parts of the frame for predicting the output. In contrast to these models, the Transformer model can have several encoders and decoders, and each of them contains its own internal attention mechanism.

An encoder block consists of sets of encoders; as a study, 6 coders are usually taken, which are located one above the other. The number of encoders is not fixed, it is possible to experiment with an arbitrary number of encoders in a block. All encoders have the same structure but different weights. The input of the encoder receives extracted feature vectors from the audio signal, obtained using Mel-frequency cepstral coefficients or convolutional neural networks. Then the first encoder transforms these data using self-attention into a set of vectors, and through the feed forward ANN transmits the received outputs to the next encoder. The last encoder processes the vectors and transfers the data of the encoded functions to the decoder block.

A decoder block is a set of decoders, and their number is usually identical to the number of encoders. Each part of the encoder can be divided into two sublayers: the input data entering the encoder first passes through the multi-head attention layer, which helps the encoder look at other words in the incoming sentence during encoding of a particular word. The output of the inner multi-head attention layer is sent to the feed-forward neural network. The exact same network is independently applied to each word in the sentence.

The decoder also contains two of these layers, but there is an attention layer between them that helps the decoder focus on significant parts of the incoming sentence, as is similar to the usual attention mechanism in seq2seq models. This component will take into account previous characters/words and, based on these data, outputs the posterior probabilities of the subsequent character/words.

### Self-attention mechanism

The Transformer model includes Scaled Dot-Product Attention^10^. The advantage of self-attention is fast calculation and shortening of the path between words, as well as potential interpretability. This attention includes 3 vectors: queries, keys and values, and scaling (7):

7 $$Attention\left({U}^{Q},{U}^{K}, {U}^{V}\right)=softmax\left(\frac{Q,K, V}{\sqrt{{d}_{attention}}}\right)V$$

These parameters are considered useful for calculating attention. Multi-head attention combines several self-attention maps into general matrix calculations (8):

8 $$MultiHeadAttention\left(Q,K, V\right)=Concat({s}_{1}, \dots , {s}_{h}){U}^{head}$$

Here ${s}_{h}=Attention(Q{W}_{h}^{Q}, K{W}_{h}^{K}, V{W}_{h}^{V})$. *h* is the amount of attention in the layer, $Q{W}_{h}^{Q}, K{W}_{h}^{K}, V{W}_{h}^{V}, {s}_{h}$ – trained weight matrices.

The multi-head attention mechanism can be used as an optimization problem. Using this mechanism, you can bypass problems associated with unsuccessful initialization, as well as improve the speed of training. In addition, after training, you can exclude some parts of the heads of attention, since these changes will not affect the quality of decoding in any way. The number of heads in the model is designed to regulate attention mechanisms. In addition, this mechanism helps the network to easily access any information, regardless of the length of the sequence, because this is done easily, regardless of the number of words in the set.

**Figure 2:**
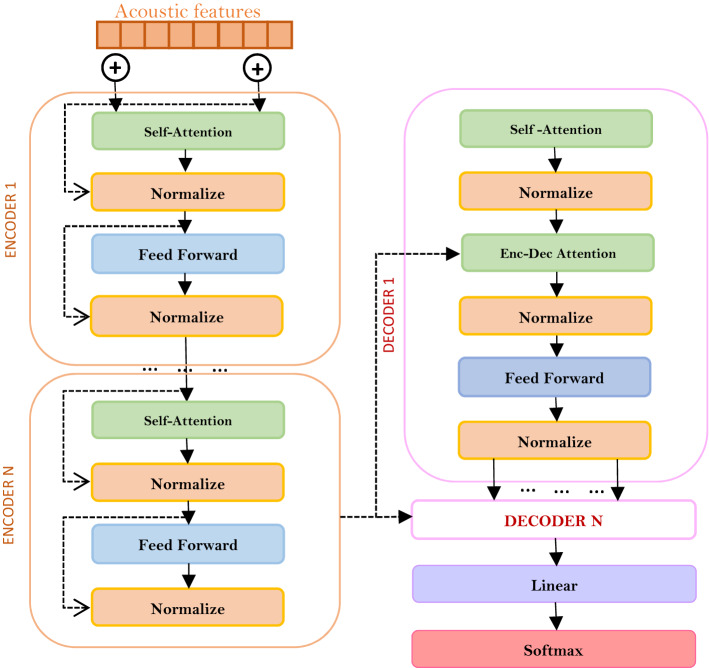
Transformer Model.

In the Transformer architecture, you can see the Normalize element, which is necessary to normalize feature values, since after using the attention mechanism, these values can have different values. As a normalization, the Layer Normalization method is usually used (Fig. 2).

The outputs of several heads can also be different, and in the final vector the spread of values can be large. To prevent this, an approach has been proposed^11^ where values at each position are converted with a two-layer perception. After applying the attention mechanism, the values are projected to a larger dimension using the trained weights, where they are then transformed by the nonlinear activation function ReLU, and then these values are projected to the original dimension, after which the next normalization occurs.

### Proposed model

Typically, Connectionist temporal classification (CTC) is used as a loss function to train recurrent neural networks to recognize input speech without pre-aligning the input and output data^11^. To achieve high performance from the CTC model, it is necessary to use an external language model, since direct decoding will not work correctly. In addition, the Kazakh language has a rather diverse mechanism of word formation, which the use of language mode contributes to an increase in the quality of recognition of Kazakh speech.

In this work, we will jointly use the Transformer and CTC models with LM. The use of LM CTC in decoding results in rapid model convergence, which reduces the amount of time to decode and improves system performance. The CTC function, after receiving the output from the encoder, finds the probability by formula 9 for arbitrary alignment between the encoder output and the output symbol sequence.

9 $${P}_{CTC}\left(W|Encoder\left(x\right)\right)= \sum_{\gamma \epsilon {R}^{-1}(y)}p(\gamma |x)$$

Here $x$ is the output vector of the encoder, *R* is an additional operator for removing blank spaces and repeated symbols,$\gamma$ is a series of predicted symbols. This equation determines the sum of all alignments using dynamic programming, and helps to train the neural network on unlabeled data.

**Figure 3:**
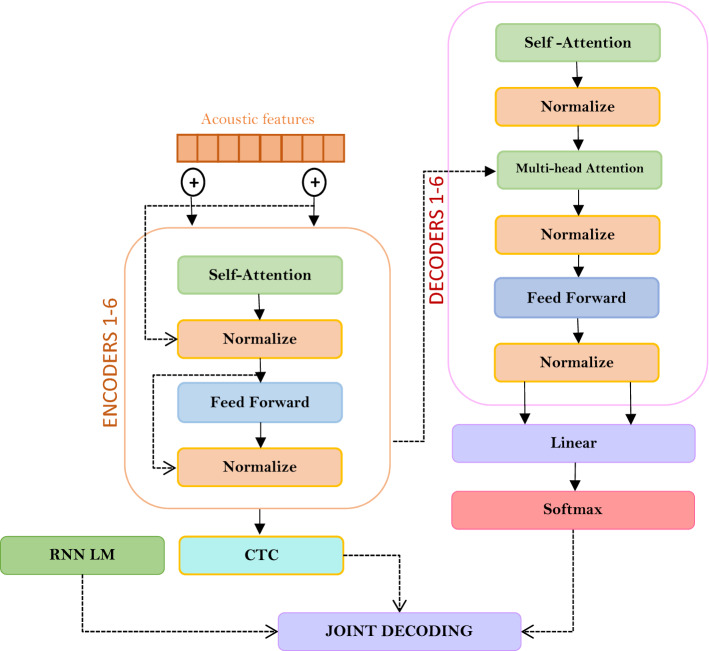
The structure of our model.

The general structure of the resulting model is shown in Fig. 3.

During training, the multi-task loss method was used to bring the general formula for combining probabilities according to the negative logarithm, as presented in^10^.

Thus, the resulting model can be represented by the following expression (10):

10 $$P\left(w|x\right)= \lambda {P}_{Transformer}+ \left(1-\lambda \right){P}_{CTC}$$

where $\lambda$—configurable parameter and satisfies the condition—$0\le \lambda \le 1$.

The following additions have been included to improve model performance:

(1) Using a character-level language model in feature extraction. Convolutional neural networks were used to extract features. To extract high-dimensional features from the audio data, we first wrap all the network parameters under the last hidden CNN layer. Softmax was used as an activation function. Next, a maxpooling layer was added to eliminate noise signals and reduce noise with dimensionality reduction. This layer is needed to reduce the size of the collapsed element into a vector. Also it helps to reduce the processing power required for data processing by reducing the dimensionality. And adaptation of training with character-level language model, without disturbing the structure of the neural network during training, allows us to preserve maximum non-linearity for subsequent processing. Thus, our extracted features are already high-level, and there is no need to map these raw data to phonemes.

(2) Application of a language model at the level of words and phrases when decoding together with CTC.

To measure the quality of the Kazakh speech recognition system, the following parameters were used: CER—the number of incorrectly recognized characters, because characters are the most common and simple output units for generating texts; and based on the word error rate (WER)^25^.

## Experiments and results

### Dataset

To train the Transformer, Transformer + CTC models with LM and without LM, it was decided to divide the corpus of 400 h of speech into two parts: 200 h of "pure" speech and 200 h of spontaneous telephone speech. This corpus was assembled in the laboratory "Computer Engineering of Intelligent Systems" IICT MES RK^13,26^. When creating the corpus, various types of speech were taken into account: prepared (reading), spontaneous. In the corpus, sound files are divided into training and test parts, these are 90% and 10%, respectively.

The pure speech database consists of recordings of 380 speakers, native Kazakh speakers of different ages and genders, as well as speech data from artistic audiobooks and audio data of news broadcasts. The voice acting and recording of each speaker took about 40–50 min. For the text, sentences with the richest phoneme of words were selected. Text data was collected from news sites in the Kazakh language, and other materials were used in electronic form in the Kazakh language.

To record the speakers, students, doctoral students and undergraduates were involved as a scientific practice, as well as employees of the institute, colleagues from different parts of the country, as well as acquaintances and relatives. Recording the voice-overs took about a year, and experts in the field of linguists and linguistics were involved to evaluate and review the corpus in order to ensure high quality.

Recordings of telephone conversations were provided by the telecommunications company for scientific use only. Transcribing of telephone conversations was carried out on the basis of the developed methodology for the compilation of texts, since this speech is spontaneous, and may contain information in a foreign language, and also speech may contain various kinds of speech noises, non-speech noises, such as a tone dial signal, a telephone beep, sounds resembling a blow, a click, as well as sounds that serve to think about the next statement. In addition, there may be slurred speech, overlapping of several speakers, etc. It should be noted that the selection of text arrays with predetermined statistical requirements for the contextual use of phonemes is a very time-consuming task and took quite a long time. This process took 5–6 months and is still ongoing. it was necessary to check not only the speech data, but also the correctness of the transcription of the data.

The speech recognition system does not require the creation of a dictionary at the phoneme level, it is enough to have audio data with text data.

After the above works, one of the important elements was created—a vocabulary base for the speech recognition system (10,805 non-repeating words). All recorded texts are collected in one file and repeated words have been removed. Once sorted alphabetically.

The audio data were in .wav format. All audio data have been converted to a single channel. The PCM method was used to convert the data into digital form. Discrete frequency 44.1 kHz, 16-bit.

The PyTorch toolkit was used for the Transformer models. The experiments were carried out on a server with eight AMD Ryzen 9 GPUs with a GeForce RTX3090. The datasets were stored on 1000 GB SSD memory to allow faster data flow during training.

### Results

To optimize the model, a gradient descent optimizer based on Adam^27^ was used with optimal parameters β_1_ = 0.8, β_2_ = 0.95 и ϵ = 10^−4^, which leads to an increase in the learning rate^10^. To improve the model, parameter values during training that affect model learning qualities were configured. At the training stage, 3 regularization methods were used, as indicated in^10^, these are residual dropout, level normalization and label smoothing. Residual dropout is applied before data normalization with a factor equal to 0.3 and then normalization is applied. The label smoothing method was applied during training with a parameter of 0.1. These regularization techniques improve the accuracy of the system's metrics and keep training from overlapping the training set. The proposed in^28^ method was used to initialize the Transformer weights and as a study, 6 encoders and 6 decoders were installed, which are located one above the other. All configurable parameters were set the same for the two datasets. The packet size was fixed at 64. For the CTC, the interpolation weight was set to 0.2 and consists of a directional six-layer BLSTM with 256 cells in each layer. The beam research width at the decode stage is 15. The language model contains two 1024-unit LSTM layers and it was trained with crated vocabulary base for speech recognition system. The model has been trained for 45 epochs.

The following tables (Tables 1,2) show the results for CER and WER of the built models on two databases (dataset). Experiments were carried out to recognize Kazakh speech using different models.

**Table 1:** Model results on database 1—Read speech.

Model	CER	WER
CTC LM	8.5	16.9
Transformer	7.6	14.1
Transformer + CTC decode	6.2	13.5
Transformer + CTC LM	3.7	8.3

**Table 2:** Model results on database 2—Conversational telephone speech.

Model	CER	WER
CTC LM	11.2	18.5
Transformer	15.9	21.2
Transformer + CTC decode	10.5	17.4
Transformer + CTC LM	9.6	15.8

The model trained on a pure data set showed competitive results only with the use of an external language model. In Table 1, it can be seen that the Transformer model with CTC works well with and without the use of the language model and achieved a CER of 6.2% and a WER of 13.5%. The integration of an external language model made the system heavier, but significantly reduced the CER and WER rates by 3.7 and 8.3%, respectively.

**Figure 4:**
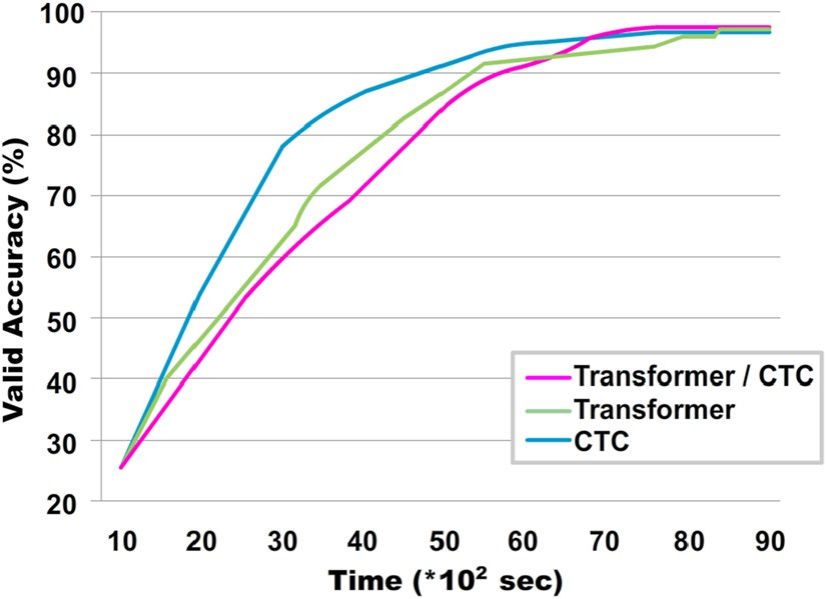
Comparing curves when training a model on Read speech.

**Figure 5:**
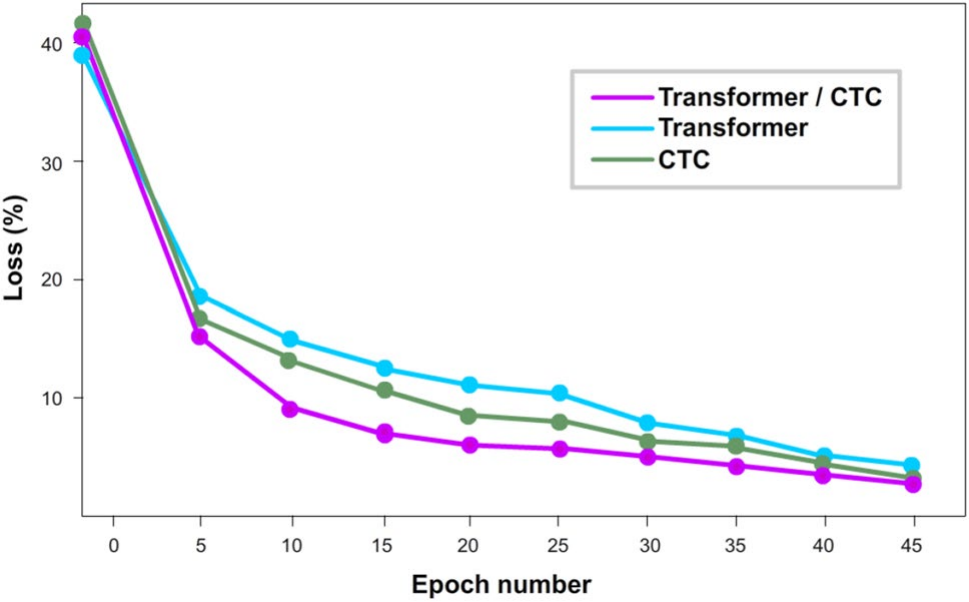
Comparative graph of Transformer + CTC and other models on the test set on Read speech.

As can be seen from the tables, the Transformer + CTC LM model shows the best result on two databases. In addition, the Transformer model with CTC learned faster and converged quickly compared to other models than without it (Figs. 4,5).

The resulting model was also easier to integrate with LM. With the help of the CTC, the obtained data were aligned.

The results obtained during the experiment prove the effectiveness of the joint use of the CTC with the E2E language model and showed the best result on all datasets in Kazakh. In addition, adding a CTC to our model generally improves the performance of the system. In the future, it is necessary to expand our speech corpus and improve CER and WER.

## Discussion

To improve the performance of these metrics, a language model trained on the basis of RNN was integrated into the model. This is the only way to achieve good results in our case. In addition, if you conduct additional experiments with a corpus that has more volume, then this addition can also affect the quality of recognition. However, increasing the amount of data for training will probably not solve the problem just like that. There are a large number of dialects and accents of Kazakh speech. It is not possible to collect enough data for all cases.

Speech recognition systems make many more errors as noise increases. This can be noticed on the basis of experiments related to the recognition of conversational telephone speech (Table 2). The model cannot simultaneously recognize 2 people who are talking at the same time, this leads to the overlap of voice data. The issues of diarization and separation of sources and the indicators for determining semantic errors have not been resolved.

Transformer takes into account the entire context and learns the language model better, and CTC helps the model learn to produce recognition that is optimally aligned in time with the recording. This architecture can be further adapted for streaming speech recognition.

## Conclusion

In this paper, the Transformer architecture for automatic recognition of Kazakh continuous speech was considered, which uses self-attention components. Despite the multiple model parameters that need to be tuned, the training process can be shortened by parallelizing the processes. The combined Transformer + CTC LM model showed better results in Kazakh speech recognition in terms of character and word recognition accuracy, and reduced these figures by 3.7 and 8.3%, respectively, than using them separately. This proves that the implemented model can be applied to other low-resource languages.

In further research, it is planned to increase the speech corpus for the Kazakh language to conduct experiments on the implemented model, and it is also necessary to make significant improvements to the Transformer model to reduce word and symbol errors in the recognition of Kazakh continuous speech.

## Data Availability

Not applicable.
